# Testing the bipolar assumption of Singer-Loomis Type Deployment Inventory for Korean adults using classification and multidimensional scaling

**DOI:** 10.3389/fpsyg.2023.1249185

**Published:** 2024-01-31

**Authors:** Sangin Lee, Jongwan Kim

**Affiliations:** Psychology Department, Jeonbuk National University, Jeonju, Republic of Korea

**Keywords:** Singer-Loomis Type Deployment Inventory, Jungian personality, bipolar assumption, multidimensional scaling, classification, intersubject correlation

## Abstract

In this study, we explored whether the Korean version of Singer Loomis Type Deployment Inventory II (K-SLTDI) captures the opposing tendencies of Jung’s theory of psychological type. The types are Extroverted Sensing, Extroverted Intuition, Extroverted Feeling, Extroverted Thinking, Introverted Sensing, Introverted Intuition, Introverted Feeling, and Introverted Thinking. A nationwide online survey was conducted in South Korea. We performed multidimensional scaling and classification analyses based on 521 Korean adult profiles with eight psychological types to test the bipolarity assumption. The results showed that the Procrustes-rotated four-dimensional space successfully represented four types of opposing tendencies. Moreover, the bipolarity assumption in the four dimensions of Jungian typology was tested and compared between lower and higher psychological distress populations via cluster analysis. Lastly, we explored patterns of responses in lower and higher psychological distress populations using intersubject correlation. Both similarity analyses and classification results consistently support the theoretical considerations on the conceptualization of Jung’s type in independent order that the types could be derived without bipolar assumption as Singer and Loomis expected in their Type Development Inventory. Limitations in our study include the sample being randomly selected internet users during the COVID−19 pandemic, despite excellence in the use of the internet in the general Korean population.

## 1 Introduction

The bipolar assumption in Jung’s theory of psychological types and its measure has been challenged (Loomis and Singer, 1980; Girelli and Stake, 1993; Arnau et al., 2000; Cook, 2003; Davis and Mattoon, 2006; Hernández-Hernández et al., 2017). The bipolar assumption presumes theoretical oppositions in attitudes (extraversion, E vs. introversion, I) and functional types in pairs of rational, also called judging (feeling, F vs. thinking, T), and irrational or perceiving (sensation, S vs. intuition, N) functions (Jung, 1971; Loomis and Singer, 1980; Garden, 1991). Jung (1971) also mentioned the deliberate exclusion between rational and irrational functions and the development of rational repressing irrational functions. According to Jung (1971), individuals rely most heavily on a ‘superior type’ relative to others while its dimensional opposite, ‘inferior function’, was thought to be repressed. Jung specified that the superior and inferior functions were expected to be members of the same function pairs, and these oppositions were bipolar in nature (Loomis and Singer, 1980).

To measure these functions in typology, Gray-Wheelwrights Jungian Types Survey (GW-JTS) and Myers-Briggs Type Indicator (MBTI) were developed and well used worldwide, but their forced-choice dichotomic format in measuring the bipolar assumption in psychological types has long been challenged and questioned (Loomis, 1982; Girelli and Stake, 1993; Turcu and Minulescu, 2019) for producing spurious negative correlations among items (Kerlinger, 1986), and their scoring system relying on this categorical approach results in distorting Jung’s theory and application (Comrey, 1983; Carlson, 1989; Healy, 1989; McCrae and Costa, 1989; McCaulley, 1991; Merenda, 1991; Girelli and Stake, 1993; Harvey et al., 1995; Garden, 1997; Spoto, 2021). Complementary to these previous inventories, Singer and Loomis developed a Singer–Loomis type development inventory, SL-TDI (Singer, 1996), to measure these functions and Jung’s psychological types with a Likert scale-based construct. The reliability and validity of SL-TDI are tested by various scholars and some observed evidence in the spectrum of the types (Loomis and Singer, 1980; Arnau et al., 2000; Capraro and Capraro, 2002; Davis and Mattoon, 2006; Park, 2013; Sato, 2017; Minulescu, 2019; Turcu and Minulescu, 2019). There was mixed evidence for these categorical approaches that the dimensional approach of types in a continuous spectrum with opposing ends was hypothesized and tested using taxometric analysis (Arnau et al., 2003). The results supported the dimensionality of Jungian psychological typology, allowing a range of strength in the type preference that Jung has mentioned (Jung 1971; Garden, 1991; Arnau et al., 2003). However, bipolar assumption and verification of the four dimensionalities and type dynamics in Jung’s psychological types lack empirical evidence and have not been directly tested. In this study, we employed state-of-the-art approaches called machine learning techniques developed in computer science and engineering to directly test the assumption.

Machine learning technique has been applied in various sectors including education (Halde, 2017; Luan and Tsai, 2021), economics (Blumenstock et al., 2015), medical imaging (Erickson et al., 2017), and clinical decision support systems (Beam and Kohane, 2018; Su et al., 2021). In psychology, machine learning techniques have been applied mainly in the field of cognitive neuroscience, confirming information on psychological function. Recent studies have applied personality traits to varied predictions (Connelly and Ones, 2010), including donation (Yarkoni et al., 2015), digital footprints such as Facebook likes, tweets, or profile pictures (Quercia et al., 2011; Kosinski et al., 2013; Nave et al., 2018), behavioral tendencies in consuming goods (Steenkamp and Maydeu-Olivares, 2015; Matz and Hirsh, 2020), relationship quality (Großmann et al., 2019), and movie preference (Nave et al., 2020). Traditional psychological research aims to establish the causal effects of predictor variables on outcome variables. In contrast, machine learning algorithms provide automated solutions for prediction without human intervention and aim to achieve maximal and unbiased classification accuracy. Machine learning develops and applies algorithms to understand complex data sets. In psychology, it has been utilized in complex neuroimaging data sets to unveil underlying structures. Multidimensional scaling (MDS) and classification methods are mostly used to decode neural representation in the domain of cognitive neuroscience (Baucom et al., 2012; Kim et al., 2016; Kim et al., 2017, 2020). MDS is one of the dimensionality reduction methodologies of unsupervised learning, and it investigates unknown internal structures present in data (Mossotto et al., 2017; Knox, 2018). Classification is categorized as supervised learning that tests the accuracy of an assigned class using a machine learning algorithm (Mossotto et al., 2017; Knox, 2018). These methods are more sensitive compared to traditional univariate analysis (Haynes and Rees, 2006; Norman et al., 2006; O’Toole et al., 2007). The number of papers using machine learning is growing as psychologists are also interested in the question of how well one variable predicts the other one (Rosenbusch et al., 2021).

Due to the multidimensional nature of Jung’s psychological typology, multivariate approaches from machine learning could be new and direct methods to explore Jung’s bipolar assumption. If individually collected eight types could be represented in the dimensions of Jung’s psychological types as a lower dimensional construct, then it will allow us to directly compare the representations of bipolar assumption in SL-TDI with theoretical assumption. Among the multivariate methods, MDS is a set of statistical techniques used to extract underlying dimensionality from high dimensional data (Kim et al., 2004, 2017, 2020; Shinkareva et al., 2013, 2014; Karstoft et al., 2015; Halde, 2017; Interian et al., 2017; Walsh et al., 2017; Shim et al., 2019; van Dam et al., 2019; Xing et al., 2020; Littlefield et al., 2021; Albagmi et al., 2022; Yang et al., 2022). For example, Kim and Kim (2022) performed both classification and MDS based on responses to ASMR stimuli to explore emotional structure. They found that positivity and negativity are located at extreme ends on a single dimension, supporting the bipolarity of the affective dimension. This study employed MDS and classification approaches to test the bipolar assumption in psychological typology.

Numerous empirical studies investigated the relationship between personality and health outcomes (Strickhouser et al., 2017). Some studies established that specific mental health is more related to certain personality types (Janowsky et al., 2002; Brown, 2009; Minulescu, 2019; Lester, 2021). In this study, we employed Korean Symptom Check List-95 (KSCL-95) to further explore the relationship between personality and mental health by examining patterns of psychological types according to the severity of mental health via cluster analysis and intersubject correlation analysis. KSCL-95 is a comprehensive psychological diagnostic test that can measure major clinical psychological symptoms (Shin et al., 2019; Jeong et al., 2020), which is used in psychotherapy (Kim and Lee, 2022) and clinical settings (Kim et al., 2019; Lee et al., 2022) in Korea. It is a test with only 95 items that compare to MMPI2 and MMPI2-RF and provides a concise yet comprehensive examination of mental health based on DSM 5. The present study aims to test the bipolarity assumption of the Jungian type theory in the four dimensions of Jungian typology in SL-TDI and compare it between lower and higher psychological distress populations measured by KSCL-95.

## 2 Method

### 2.1 Participants

Five hundred and thirty adults over 18 years old participated in this survey online in South Korea. Each participant was provided with a thorough written description of the experiment and signed informed consent in accordance with the Institutional Review Board at the Jeonbuk National University (2020-09-014-001). Analyses were conducted on the remaining 521 participants (385 women, mean age 26.26 SD = 9.1 range of age 18–73) due to duplicate data and missing data.

### 2.2 Scales

#### 2.2.1 Singer-Loomis Type Deployment Inventory (SL-TDI)

The Korean version of SL-TDI was first validated in 2013 (Park, 2013), and this study used the updated Korean version of SL-TDI in 2021. The SL-TDI consists of 20 hypothetical situations, each followed by a list of eight possible reactions to the situation (Singer et al., 1996). Each reaction corresponds to a combination of an introverted or extroverted orientation with each of the four functions. The respondent indicates on a five-point Likert scale how often he or she would make that response (1 is never and 5 is always). The Cronbach’s α in the present sample was 0.94 for the complete inventory and in the eight types of EF, IF, ET, IT, ES, IS, EN, and IN were, respectively, 0.74, 0.61, 0.69, 0.73, 0.71, 0.69, 0.69, and 0.76.

#### 2.2.2 Korean Symptom Check List-95

The KSCL-95 is a 95-item self-report on a four-point Likert scale (0–3), developed by modifying SCL-90-R (Derogatis and Unger, 2010) to reflect the DSM-5 of the current mental health environment and socio-cultural characteristics of South Korea (Kwon, 2015). It is a comprehensive test assessing major clinical psychological symptoms such as anxiety, depression, obsessive-compulsive behaviors, and paranoid tendencies. According to the manual, scorings are categorized into low, moderate, caution, and clinical ranges by under 40, 40 ≤ T < 60, 60 ≤ T < 70, and over 70, respectively. Higher scores reflect a higher level of psychological distress as well as a greater severity of self-reported symptoms. The Cronbach’s α in the present sample was 0.95.

### 2.3 Data analyses

#### 2.3.1 Cluster analysis

K-means cluster analysis was performed to extract clusters and categorize participants based on the KSCL-95 levels. A two-cluster solution was derived through the K-means clustering because they best conceptualized the clusters’ characteristics of high level (the higher psychological distress population) and low level (the lower psychological distress population). We tested whether the cut-off level for KSCL 95 differed significantly between the lower and higher psychological distress populations using t-tests. Differences between the two sets of K-means clusters and cut-off clusters were explored.

#### 2.3.2 Multidimensional scaling (MDS)

Multidimensional scaling was performed to find the lower dimensional representation of Jung’s typology and to test the bipolar assumption by evaluating relationships between the extracted dimensions with hypothesized design values (Table 1). Using all 160 items, bipolar assumptions in four dimensions of attitude (extroverted, E vs. introverted, I), perceiving (sensing, S vs. intuition, N), judging (feeling, F vs. thinking, T), and lifestyle (perception, P vs. judgment, J) were tested by the representation of the eight psychological types on the multidimensional space with MDS. We performed MDS for each cluster identified by K-means clustering in the following steps. We ran MDS using a 160 × 160 correlation matrix. Then, the derived 4-dimensional MDS solution was Procrustes rotated to design values (Table 1), and the point-biserial correlations were performed to test the significance of the relationship between the solutions and design matrix values. These MDS procedures were repeated for the depicted two populations from the cluster analysis to explore the differences between the lower and higher psychological distress populations.

**Table 1:** Design matrix of SL-TDI.

Dimension ID	Dimensions	Design values					
		E	I	S	N	F	T
1	Attitude E vs. I	1	-1	0	0	0	0
2	Lifestyle P vs. J	0	0	1	1	-1	-1
3	Perception S vs. N	0	0	1	-1	0	0
4	Judgment F vs. T	0	0	0	0	1	-1

#### 2.3.3 Classifications

Another technique to test the bipolar assumption was classification. We collected our data in a structure of SL-TDI items * survey participants. We used participants as a feature to predict the item types. Classifications included four 2-way classifications (E vs. I, J vs. P, S vs. N, and F vs. T) and 8-way classification. All 160 items of SL-TDI were used for E vs. I and J vs. P classifications, and 80 items for S vs. N and F vs. T classifications. In each of the cross-validation folds, one of the items was left out as a test data set when the classifiers were trained on the remaining items as training sets. The Support Vector Machine (SVM) classifier was constructed from the training set and applied subsequently to the unused test set. Classification accuracies were computed based on the average classification accuracy across 160 items for E vs. I and J vs. P and 80 items for S vs. N and F vs. T classification cross-validation folds. We tested if classification accuracies were significantly higher than chance levels. Significance testing was conducted with a one-sample t-test to evaluate if the classification accuracies were significantly above the chance level (0.5 for 2-way and 0.125 for 8-way).

#### 2.3.4 Intersubject correlation

Intersubject correlation (ISC) analysis was conducted to examine the consistency of the responses between individuals for each cluster (Kim et al., 2022). Three different ISC analyses were computed for each population and the differences between the groups were tested using t-tests. First, ‘total ISC’ compared one individual subject to the rest of all the participants. Ratings of each individual and the average of the rest of the participants were correlated, yielding 521 individual ISCs. Then, ISCs were separated into the lower and higher psychological distress populations to test the difference between the two clusters. The second ISC analysis, ‘within population ISC’, was calculated to compute ISC within each of the lower and higher psychological distress clusters. The within-population ISC was computed only within each of the two clusters. We correlated one individual subject from a cluster to the belonging cluster with an average of the rest of the participants in the cluster. For example, one normal individual was compared with the rest of the normal population. Third, the ‘cross population ISC’ analysis was derived by correlating one subject from one cluster with the average of the other cluster. We used the leave-one-out approach to compute all three different ISCs at the individual subject level.

## 3 Results

### 3.1 Demographic

Detailed demographics are provided in Table 2. For the demographic data, no significant differences were observed in terms of gender (*t* = 0.679, *p* = 0.498, two-sample t-test) and age (*F* = 1.354, *p* = 0.232, one-way ANOVA) for the SL-TDI test.

**Table 2:** Demographics of participants.

	*N*	%	cum%
10’s	91	17.5	17.5
20’s	318	61.0	78.5
30’s	75	14.4	92.9
40’s	11	2.1	95.0
50’s	18	3.4	98.4
60’s	6	1.2	99.6
70’s	2	0.4	100.0
Male	136	26.1	26.1
Female	385	73.9	100.0
Total	521	100.0	100.0

### 3.2 Testing bipolar assumption

**Figure 1:**
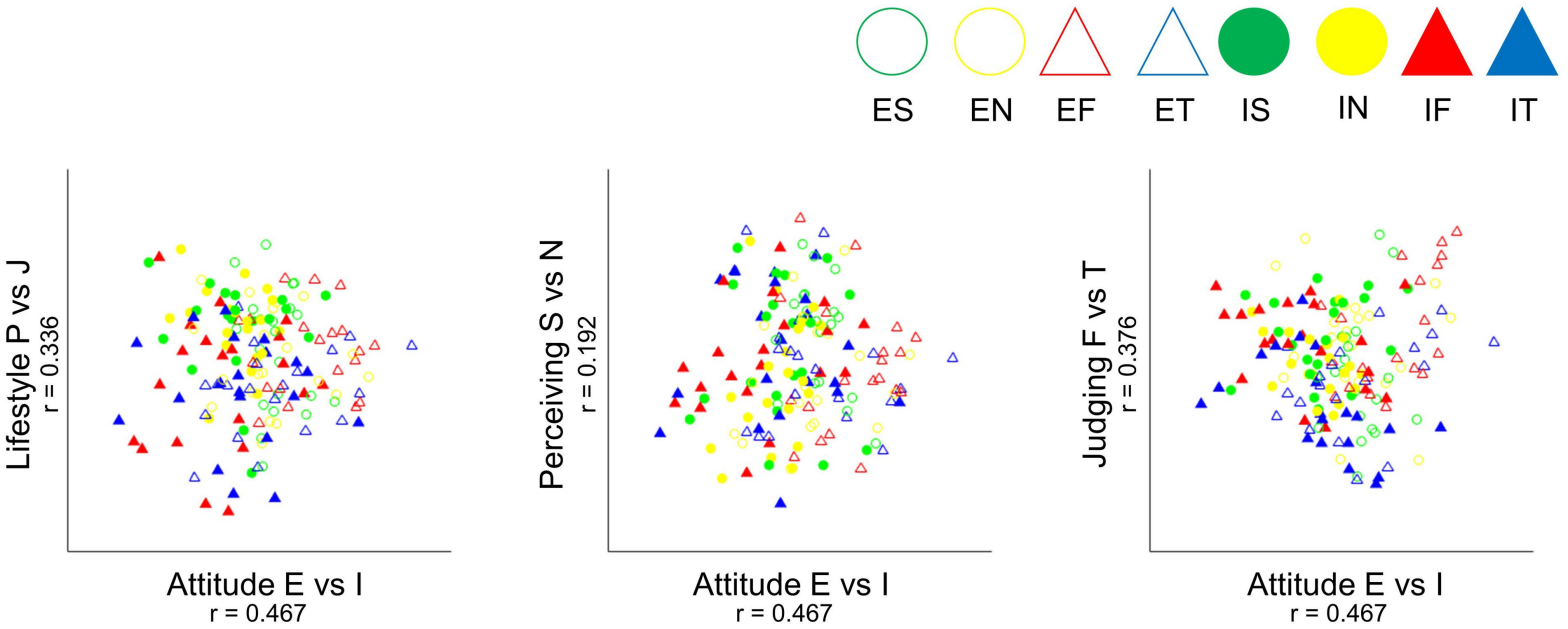
Results of multidimensional scaling with procrustes rotation to design matrix (Table 1). The three plots represent multidimensional relations of Jungian typology and its bipolarity. The left plot indicates the x-axis of attitude (E vs. I) in relation to the y-axis of lifestyle (P vs. J) dimension. The correlation of rotation and the design matrix resulted in 0.467 for attitude (E vs. I) and 0.336 for lifestyle (P vs. J). The x-axis of attitude (E vs. I) is shown in relation to the y-axis of perceiving (S vs. N) dimension in the center plot and judging (F vs. T) dimension for the right plot. The correlation of rotation and the design matrix resulted in 0.467 for attitude (E vs. I), 0.336 for lifestyle (P vs. J), 0.192 for perceiving (S vs. N), and 0.376 for judging (F vs. T). E, extroverted; I, introverted; P, perceiving; J, judging; S, sensing; N, intuition; F, feeling; T, thinking.

We examined whether the patterns of self-reported SL-TDI results assessing Jungian typology represented identifiable bipolar assumptions of the Jungian personality theory. The bipolar assumption in four dimensions of the Jungian psychological types of attitude (E vs. I), lifestyle (P vs. J), perceiving (S vs. N), and judging (F vs. T) functions at their bipolar opposites regarding the psychological process involved are represented in Table 1. The results showed that Pearson correlations between rotated MDS solutions and design matrices for four dimensions were all above the critical value of 0.157, indicating a significant identification of bipolar assumptions (Figure 1). More specifically, the introverted and extroverted attitudes were well separated in Dimension 1; sensation and intuition were located above, and feeling and thinking were located toward the bottom of the plot. The attitude dimension where extroverted (E) and introverted (I) were bipolar opposites had 0.47, the function dimension of judging with opposites of feeling (F) and thinking (T) had 0.376, the lifestyle with opposites of perceiving (P) and judging (J) had 0.336, and the perceiving functional dimension with opposites of sensation (S) and intuition (N) showed 0.192, with respect to highest correlation distance.

**Figure 2:**
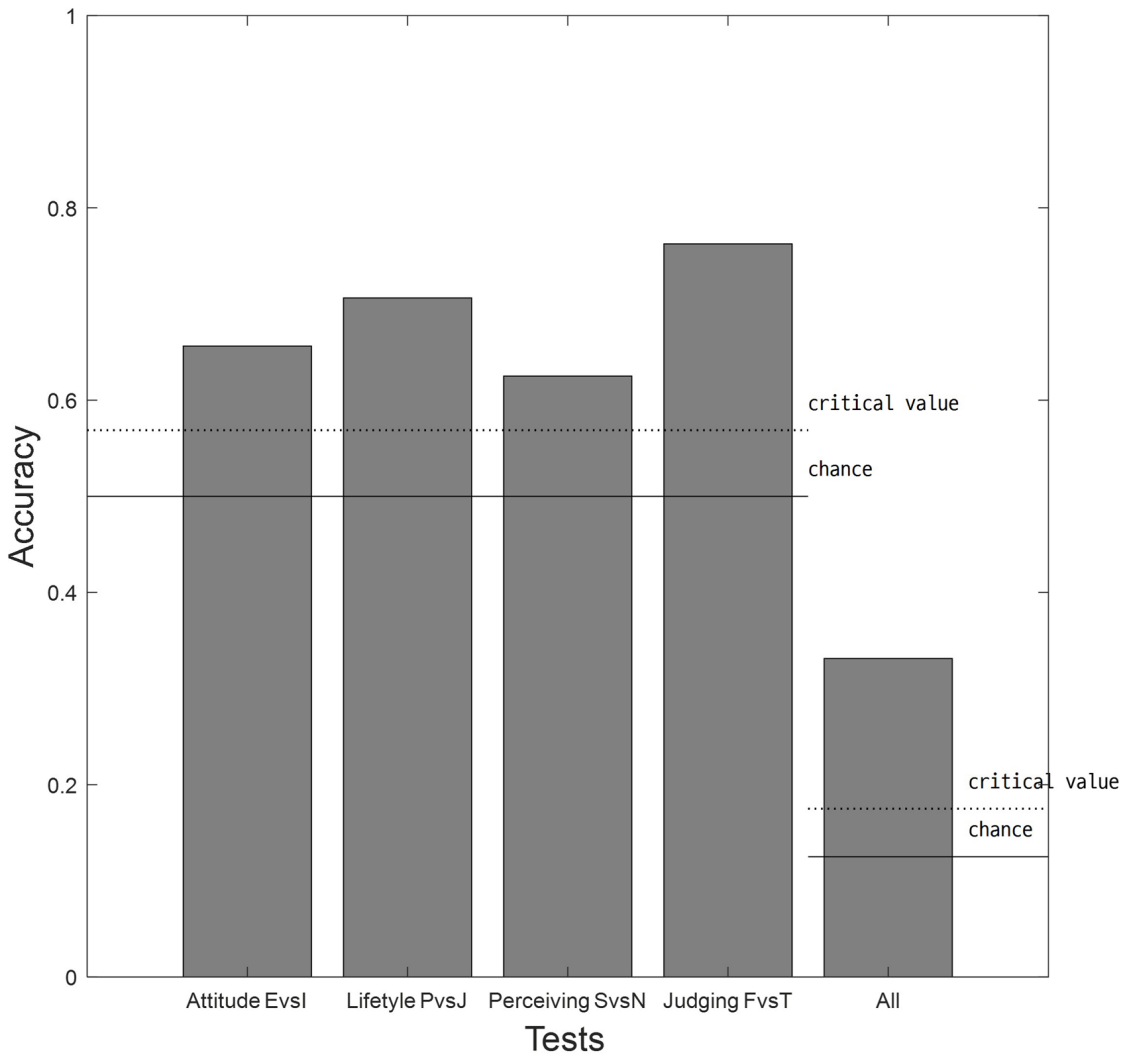
Results of classifications of bipolar assumptions. Each bar indicates the accuracy of bipolar assumption in the four dimensions of attitude (E vs. I), lifestyle (P vs. J), perceiving (S vs. N), and judging (F vs. T) function and SL-TDI. E, extroverted; I, introverted; P, perceiving; J, judging; S, sensing; N, intuition; F, feeling; T, thinking.

Classification analyses revealed the above-chance accuracies over its critical value of 0.5687 for the 2-way classifications and 0.175 for the 8-way classifications. Then, we also tested bipolar assumption using classification for identified clusters. The results revealed that classification accuracies for bipolar assumption with a total of 521 participants ranged from 0.625 to 0.763, with all accuracies significantly greater than chance (*p*s < 0.05) (Figure 2).

#### 3.2.1 Testing bipolar assumption for each cluster

##### 3.2.1.1 Cluster analysis

Cluster analysis was carried out to differentiate between lower and higher psychological distress populations. We selected two cluster solutions. Cluster 1 denoted 390 participants with low values of all sub-measures of KSCL-95, which is the lower level of psychological distress population participants (average score of 47.70 and 74.86% total participants). Cluster 2 had high values of KSCL-95, which is the higher level of psychological distress population with 131 participants (average score of 62.43 and 25.14% total participants). The selected two cluster solutions representing lower and higher levels of psychological distress populations derived through the K-means clustering based on KSCL-95 levels are shown in Supplementary Figure S1. Independent measures ANOVA confirmed a significant difference between the two K-means cluster solutions for all disorders measured in KSCL-95, *p*s < 0.05.

### 3.3 Comparison between the two clusters

#### 3.3.1 Multidimensional scaling analysis and classification

**Figure 3:**
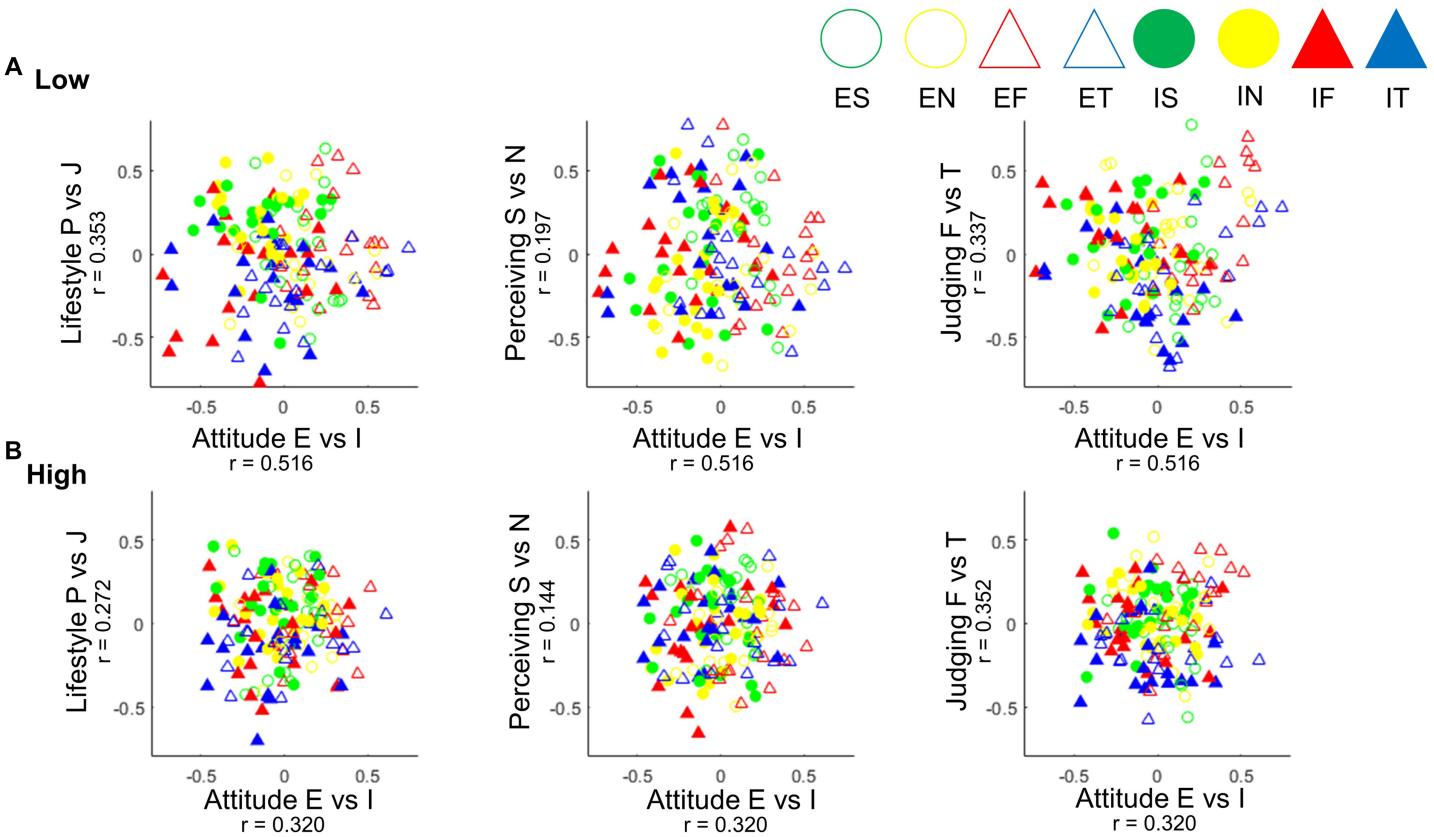
Results of MDS based on the correlation between design matrix (Table 1) and MDS solution coordinates after Procrustes rotation for lower and higher psychological distress clusters. **(A)** The Pearson correlation of rotation and the design matrix for the lower psychological distress population resulted in 0.516 for attitude (E vs. I), 0.353 for lifestyle (P vs. J), 0.197 for perceiving (S vs. N), and 0.337 for judging (F vs. T). **(B)** For the higher psychological distress population, the Pearson correlation of rotation and the design matrix were 0.32 for attitude (E vs. I), 0.272 for lifestyle (P vs. J), 0.144 for perceiving (S vs. N), and 0.352 for judging (F vs. T). E, extroverted; I, introverted; P, perceiving; J, judging; S, sensing; N, intuition; F, feeling; T, thinking.

We compared representations of the bipolar assumptions in all four dimensions of the Jungian psychological types of attitude (E vs. I), lifestyle (P vs. J), perceiving (S vs. N), and judging (F vs. T) functions from the two clusters. The SL-TDI was represented in lower dimensional spaces using MDS analysis. The results showed that Pearson correlations between rotated MDS solutions and design matrices (Table 1) for four dimensions were all above the critical value of 0.157, indicating a successful identification of bipolar assumptions for both clusters (Figure 3).

There were significantly greater correlations for the lower psychological distress population in attitude (E vs. I), lifestyle (P vs. J), perceiving (S vs. N), and judging (F vs. T) function dimensions, representing better distinction in the opposites of the psychological types than those of higher psychological distress population. The difference between the two clusters in the judgment functional dimension was not significant (*z* = 0.43). The higher psychological distress population did support bipolar assumptions in attitude (E vs. I), lifestyle (P vs. J), and judging (F vs. T) functions. However, the perceiving (S vs. N) function was not represented, *r* = 0.144, *p* > 0.05.

**Figure 4:**
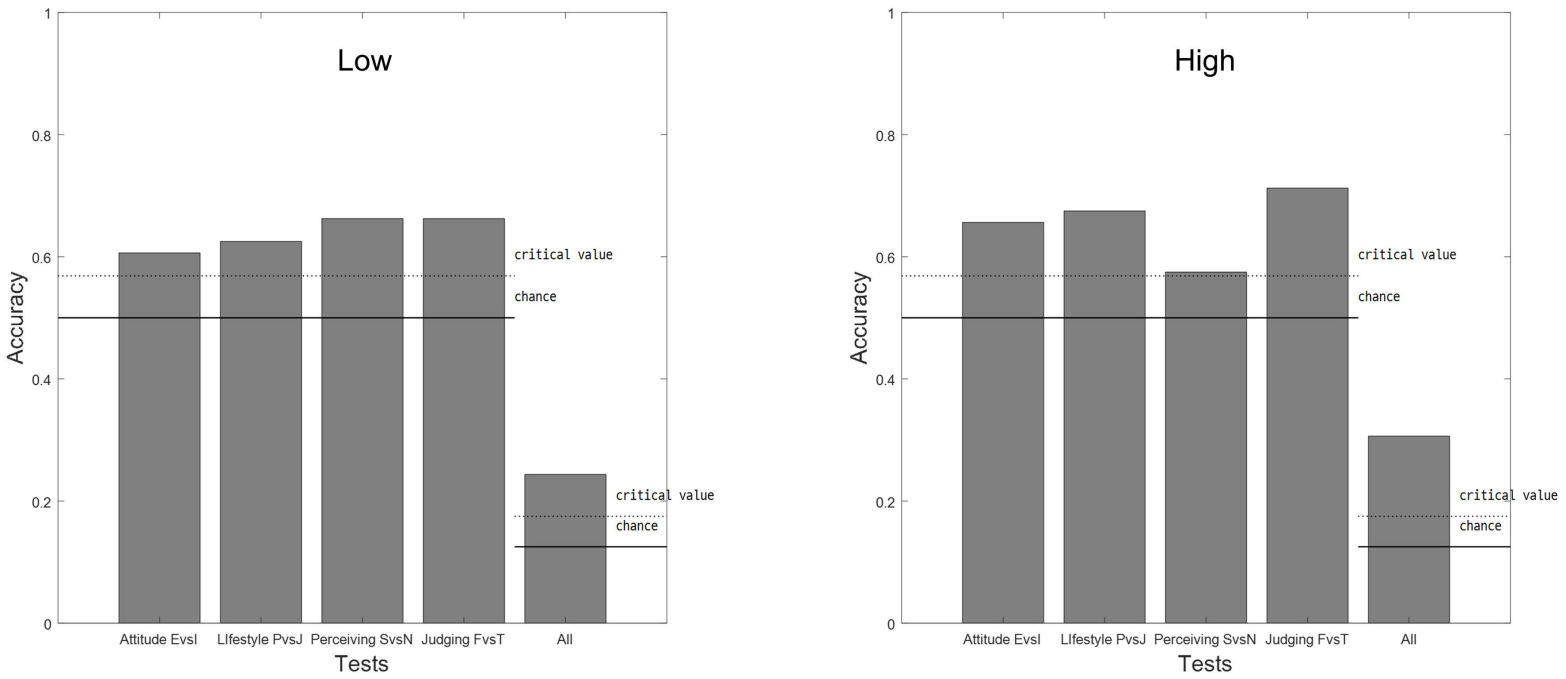
Results of classifications for each cluster. Each bar indicates accuracies across bipolar assumptions and SL-TDI for the lower psychological distress population (left) and higher psychological distress population (right). A description of the selection methods can be found in the text. E, extroverted; I, introverted; P, perceiving; J, judging; S, sensing; N, intuition; F, feeling; T, thinking.

Then, we also tested bipolar assumption using classification for each of the identified clusters (Figure 4). Classification results show that all bipolar assumptions (E vs. I, J vs. P, S vs. N, and F vs. T) revealed significant accuracy over the chance for both lower and higher psychological distress populations. The lower psychological distress population shows the classification accuracies for bipolar assumptions in attitude (E vs. I), lifestyle (P vs. J), perceiving (S vs. N), and judging (F vs. T) functions at 0.600, 0.631, 0.663, and 0.663, respectively. The classification accuracy of 0.575 for perceiving (S vs. N) from the higher psychological distress population was close to the critical value of 0.568, which is consistent with the results of the MDS.

#### 3.3.2 Intersubject correlation

We examined the patterns of the two cluster groups via intersubject correlation analyses. Within-population ISC represented that each population had a relatively constant pattern in their responses to KSCL-95 at the mean ISCs of 0.40 and 0.36 for the lower and higher psychological distress populations, respectively. Then, the cross-population ISC results of 0.011 and 0.0166 ISC for the lower and higher psychological distress populations also support that the two populations had distinct pattern characteristics in their response to KSCL. The lower psychological distress population had significantly more constant intersubject correlations (*M* = 0.363), while the higher psychological distress population expressed less in common with the pattern of the total participants (*M* = 0.159).

Our examination of the ISC of SL-TDI also indicated a significant difference between the two clusters. The ISC analysis of the lower psychological distress population to the total participants shows a mean ISC of 0.465 while the higher psychological distress population shows 0.405; there was a significant difference in the two clusters, *t* (519) = 5.07, *p* < 0.001. The within-population ISC indicated the lower psychological distress population has a mean of 0.466 and the higher psychological distress population has a mean of 0.410, and there was a significant difference in the ISC results of two clusters within the population, *t* (519) = 4.67, *p* < 0.001. The cross-population ISC reveals the mean ISC of 0.439 for the lower and 0.397 for the higher psychological distress population. The t-test indicated a significant difference in the ISC of the two when one individual was compared to the pattern of the other cluster group, *t* (519) = 3.59, *p* < 0.001. These results suggest possible personality pattern differences between the two clusters.

## 4 Discussion

In this study, we directly tested the bipolar assumption of Jung’s psychological typology in SL-TDI using machine learning methods and also compared the lower and higher psychological distress populations. The bipolar assumptions in all four dimensions of the Jungian psychological types were tested. The MDS and classification successfully revealed that the SL-TDI is a valid psychometric assessment for bipolar assumption in Jung’s psychological typology. Moreover, the bipolar assumptions were tested and compared between the lower and higher psychological distress populations. Examination of the homogeneity of the SL-TDI pattern revealed that the lower psychological distress population showed higher ISCs in within- and between-group comparisons than the higher psychological distress population. This suggests that the higher psychological distress group was more heterogeneous than the general less stressed population. However, this study was conducted during COVID−19, and randomly collected sample populations with approximately three to one gender imbalance participated in the survey.

This study demonstrated successful applications of state-of-the-art machine learning techniques to survey data. This advantage of multivariate analysis includes (1) a relatively small loss of information collected from the data (Haynes and Rees, 2006), and (2) safety from committing multiple comparison errors, which increases the level of error as the number of comparisons between groups increases. The multidimensional scaling method uses similarity data to locate the items in a lower dimensional space so that they can be easily identified while maintaining the relationship between items and the structure of the data (Malhotra et al., 1996). Classification analysis is a multivariate technique that attempts to classify or predict with consideration of all measured data as features and could function as a decoding analysis (Weaverdyck et al., 2020). We employed a data-driven method, cluster analysis, to identify populations with lower and higher psychological distress, instead of using specific cut-off points. Little evidence has been found on specific psychological disorders, but overall, there was a statistically significant mean difference in KSCL-95 between the lower and higher psychological distress populations.

Several kinds of frameworks can predict individual differences in traits and behavior based on neuroimaging data, such as the multivariate-prediction method using support vector regression (Harris et al., 1996). Along with the intersubject network similarity approach (Liu et al., 2019), many studies have been suggesting the use of the multivariate technique in identifying the participants in terms of not only predicting behaviors from brain connectivity patterns but also in pure behavioral (Kim et al., 2022, Jang, in press), psychosocial profiles (Ding, 2006), or clinical data, such as PTSD (Rosellini et al., 2018; Shim et al., 2019; Wshah et al., 2019), anxiety (Carpenter et al., 2016; Anugraha and Vineetha, 2018; Muhammad et al., 2020; Xing et al., 2020), depression (Priya et al., 2020; Asare et al., 2021; Su et al., 2021; Yang et al., 2022), and suicide (Walsh et al., 2017; Littlefield et al., 2021; Schafer et al., 2021; Zheng et al., 2022). Usually, individual differences in scales are represented by a single score, the average performance, or with total scores of subscales. However, for personality-related tests, each item within the personality dimension can better represent subtle differences in behavioral tendencies and cognition than the total score (Chapman, 2007; Watson et al., 2007). The multi-sub-factor nature of the personality measure is one potential obstacle to individual prediction (Liu et al., 2019). Participants with the same sum score may still have differences in their questionnaire responses and patterns. Our approach applies to the prediction of sum scores and a pattern in the responses.

Our results indicated that classification between intuition and sense functions was not statistically significant for the higher psychological distress population; however, it was significant for the less distressed population.

There are very few studies testing dimensions with less adaptive personality traits (McCrae and Costa, 1989; MacDonald and Holland, 1993; MacDonald et al., 1994; Furnham et al., 2003) and psychological disorders (Coolidge et al., 2001; Furnham and Crump, 2014), and ours is the first to directly test the bipolarity of personality types in the population with varying levels of psychological distress. Thus, it was challenging to compare our study with previous studies. We looked into indirect evidence in previous studies, including correlation studies. Under the assumption that if bipolarity of a dimension is not apparent in the higher psychological distress population, there would be less correlation between the opposing poles of the dimension and psychological disorders, the following studies were examined for presenting less correlation with psychological disorder and bipolarity in S vs. N dimension. Our study was consistent with a previous study conducted on college students with at least one clinical score (over 60) in MMPI, in terms of their correlation results between MBTI and the clinical score (Lim et al., 2008). Out of the bipolar dimensions, S vs. N and F vs. T were not as significantly correlated with MMPI subscales as other dimensions. Furnham and Crump (2014) used the Hogan Development Survey to measure dark-side variables that depict personality disorders and the MBTI on working adults in England. Five of the 11 dark-side traits were correlated with the E vs. I dimension, none with S vs. N, seven with F vs. T, and four with the J vs. P. The replication of their study once again revealed the S vs. N dimension of the MBTI was unable to explain the aberrant dark-side personality traits or disorders, while the attitude E vs. I and judging F vs. T dimensions did (Furnham and Crump, 2014; Furnham and Furnham, 2022). Psychotherapy-referred veteran patients show a less distinctive dichotomous preference for S vs. N than other dimensions’ bipolarity in comparison to the general population (Otis and Louks, 1997). Similarly, affective disorder patients show overall ambiguity in the S vs. N dimension compared to the normative sample (Janowsky et al., 2002). Moreover, when analyzing the variance of MMPI subscale scores and MBTI dimensions, E vs. I, J vs. P, and F vs. T dimensions caused significant effects, but none of the MMPI subscales produced significant findings with S vs. N dimension independently. Similarly, Coolidge et al. (2001) reported that the dimension of S vs. N in adults was not as strongly correlated with personality disorders measured by the Coolidge Axis II Inventory (CATI) compared to other dimensions measured by MBTI (Coolidge et al., 2001). However, CATI borderline and narcissistic personality disorders were positively correlated with S vs. N dimension. Comparatively, the NEO-Personality Inventory Neuroticism scale correlates only weakly or not at all with any of the MBTI scales (McCrae and Costa, 1989; MaCDonald and Holland, 1993). Unipolar depressed patients and bipolar patients who were depressed and manic showed no significant difference in their MBTI S vs. N dimension (Janowsky et al., 1999). From these results, we speculate that this difference between the clusters may be due to difficulties of psychologically ill people. Sensing is a function that describes paying attention to the reality of one’s external environment. In contrast, intuition incorporates a sense of time and allows for hunches. We concluded that our results could be inferred as the psychologically distressed population experiencing difficulty distinguishing what is out in reality and inner hunches. Our findings also support previous studies in the significantly less distinction between intuition and sensing function from borderline personality patients than that of the normal population (Davis, 1991) and bipolar and unipolar disorder patients (Lombion-Pouthier et al., 2006; Martino et al., 2011; Lahera et al., 2016; Taalman et al., 2017). The perceiving function was also related to antisocial, borderline, passive-aggressive, sadistic, and schizotypal characteristics (Dean, 1984; Coolidge et al., 2001; Moberg et al., 2003; Rupp, 2003; Hagiya et al., 2015).

Since our survey was conducted online, more participants with easier access to the internet via electronics were included in our data. As a result, most participants were less likely to experience clinical symptoms and evident personality disorders; we characterized our participants in clusters of experiencing lower and higher psychological distress, suggesting our results generally apply to individuals with functions and less severe symptoms. Future studies could include inpatient and severe psychiatric patients or specifically diagnosed patients to further explore the matter.

The current study may be the first to directly test Jung’s psychological typology in SL-TDI as far as we know. Our overall prediction accuracy was significantly higher than chance, and the predicted Jung’s psychological typology significantly correlated with the subject’s measured typology. These findings suggest that individuals did represent all four dimensions in bipolarity assumptions.

## 5 Conclusion

Our study supports the bipolarity assumption of Jung’s psychological typology. We directly tested and demonstrated it in all four dimensions of attitude (extraversion, E vs. introversion, I) and functional types in pairs of rational (feeling, F vs. thinking, T) and irrational (sensation, S vs. intuition, N), and between judging and perceiving functions using SL-TDI. MDS and classification successfully revealed that although SL-TDI individually measures the eight psychological types, there was bipolarity in all dimensions. This was possible methodologically due to the recent and promising advance of machine learning methodology in the psychological field. Moreover, we observed possible differences in typology distribution for lower and higher psychological distress populations.

## Data availability statement

The datasets generated during and/or analyzed in the current study are not publicly available due to copyright issues related to the scales. Requests regarding the datasets should be made to the corresponding author.

## Ethics statement

The studies involving humans were approved by the Institutional Review Board at the Jeonbuk National University. The studies were conducted in accordance with the local legislation and institutional requirements. The participants provided their written informed consent to participate in this study.

## Author contributions

SL and JK contributed to the conception and design of the study and performed the statistical analysis under JK’s supervision. SL organized the database and wrote the first draft of the manuscript. All authors contributed to the manuscript revision, and read and approved the submitted version.
