# Supplementary figures and images for: Testing the bipolar assumption of Singer-Loomis Type Deployment Inventory for Korean adults using classification and multidimensional scaling
Source: Front Psychol. 2024 Jan 31;14:1249185. doi: 10.3389/fpsyg.2023.1249185 (PMC10864660; doi:10.3389/fpsyg.2023.1249185)

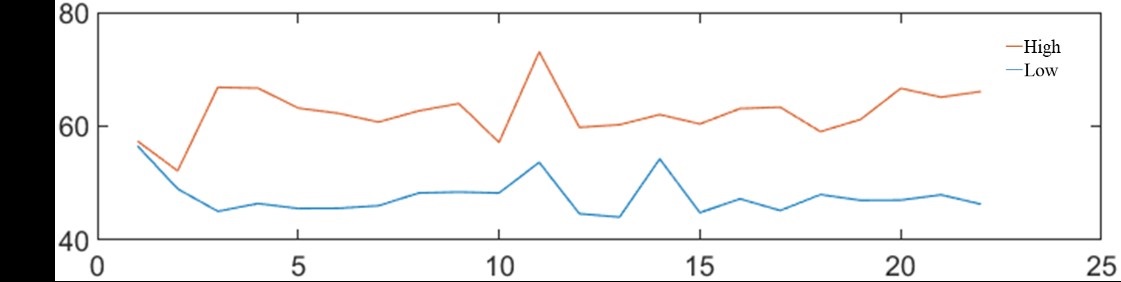

Supplement: Supplementary file 1 [file Image_1.JPEG]
